# Are "functionally related polymorphisms" of renin-angiotensin-aldosterone system gene polymorphisms associated with hypertension?

**DOI:** 10.1186/1471-2261-10-23

**Published:** 2010-06-02

**Authors:** Ines N Hahntow, Gideon Mairuhu, Irene GM van Valkengoed, Richard P Koopmans, Martin C Michel

**Affiliations:** 1 Dept. of Pharmacology & Pharmacotherapy, Academic Medical Center, University of Amsterdam, Meibergdreef 15, 1105 AZ Amsterdam, The Netherlands; 2 Dept. of Internal Medicine, Academic Medical Center, University of Amsterdam, Meibergdreef 15, 1105 AZ Amsterdam, The Netherlands; 3 Dept. of Social Medicine, Academic Medical Center, University of Amsterdam, Meibergdreef 15, 1105 AZ Amsterdam, The Netherlands; 4 Dept. of Internal Medicine, Academic Hospital, University of Maastricht, Debyelaan 25, 6202 AZ Maastricht, The Netherlands

## Abstract

**Background:**

Genotype-phenotype association studies are typically based upon polymorphisms or haplotypes comprised of multiple polymorphisms within a single gene. It has been proposed that combinations of polymorphisms in distinct genes, which functionally impact the same phenotype, may have stronger phenotype associations than those within a single gene. We have tested this hypothesis using genes encoding components of the renin-angiotensin-aldosterone system and the high blood pressure phenotype.

**Methods:**

Our analysis is based on 1379 participants of the cross-sectional SUNSET study randomly selected from the population register of Amsterdam. Each subject was genotyped for the angiotensinogen M235T, the angiotensin-converting enzyme insertion/deletion and the angiotensin II type 1 receptor A1166C polymorphism. The phenotype high blood pressure was defined either as a categorical variable comparing hypertension versus normotension as in most previous studies or as a continuous variable using systolic, diastolic and mean blood pressure in a multiple regression analysis with gender, ethnicity, age, body-mass-index and antihypertensive medication as covariates.

**Results:**

Genotype-phenotype relationships were explored for each polymorphism in isolation and for double and triple polymorphism combinations. At the single polymorphism level, only the A allele of the angiotensin II type 1 receptor was associated with a high blood pressure phenotype. Using combinations of polymorphisms of two or all three genes did not yield stronger/more consistent associations.

**Conclusions:**

We conclude that combinations of physiologically related polymorphisms of multiple genes, at least with regard to the renin-angiotensin-aldosterone system and the hypertensive phenotype, do not necessarily offer additional benefit in analyzing genotype/phenotype associations.

## Background

Genotype-phenotype association studies have become important tools to explore the pathophysiology of many disease states. They are typically based on single polymorphisms in genes of interest. In some cases multiple polymorphisms within a given gene exist in a fixed combination, i.e. as haplotypes which may exhibit stronger/more consistent associations with phenotypes than single polymorphisms [1,2].

An expansion of this thought has been based upon studies in the renin-angiotensin-aldosterone (RAAS) system. The RAAS is an important regulator of cardiovascular function and blood pressure [3,4]. It consists mainly of the angiotensin-converting enzyme (ACE) which metabolizes angiotensinogen (AGT) to form angiotensin II, which can act angiotensin II type 1 receptors (AGTR1) to mediate blood pressure elevations by mechanisms including direct effects on vascular tone and indirect effects via alterations of renal function. Thus, ACE, AGT and AGTR1 act synergistically on the phenotype of blood pressure. Each of the three corresponding genes has several polymorphisms that can be associated with altered expression or function of the corresponding gene product. While each of these polymorphisms may potentially affect the regulation of cardiovascular function by the RAAS, most previous studies have focused on one polymorphism in each of these genes, i.e. the single nucleotide polymorphism (SNP) M235T within exon 2 of the AGT gene, an insertion/deletion (I/D) polymorphism involving 287 bp in intron 16 of the ACE gene and the A1166C SNP in the 3' untranslated part of the AGTR1 gene [5]. The 235T allele of the AGT gene is associated with a stepwise increasing level of circulating angiotensinogen ("gene dose-response") [6,7]. The ACE I/D polymorphism is strongly associated with the level of circulating enzyme, with mean plasma ACE activities of DD carriers being about twice those of II subjects and heterozygotes having intermediate levels [8]. The direct functional relevance of the 1166C allele of the AGTR1 gene is less clear, but it was shown recently to attenuate the microRNA-155 binding leading to a decreased translation i.e. less receptor density in endothelial and vascular smooth muscle cells [9] and this is associated with altered serum aldosterone concentrations [10].

Accordingly, numerous studies have tested whether any of the above three polymorphisms is associated with the presence or severity of arterial hypertension (HT), almost all of them using the categorical variable HT rather than the underlying continuous variables of measured blood pressure. However, the available data remain equivocal as reports of associations have not been consistently confirmed, and even reports on inverse associations have been published [5,11]. Retrospectively, this is not too surprising as more recent genome wide analyses have not implicated any of these three gene loci in arterial hypertension [12-16]. Therefore, despite being an attractive and pathophysiologically logical concept, the existence of associations between polymorphisms in any of the three main RAAS genes and HT remains unclear. Similarly disappointing results of association studies have been found between polymorphisms of components of the RAAS and some phenotypes other than HT. However, for phenotypes related to renal function a novel concept has been proposed which has yielded stronger genotype-phenotype associations. This concept is based on the idea of synergistic effects of RAAS components and proposes that polymorphisms in each of the RAAS components, which have minor effects in isolation, may have greater effects when they occur concomitantly. Indeed a combined analysis of polymorphisms in the ACE, AGT and AGTR1 genes has shown stronger associations with proximal renal sodium handling [17] or the development of renal insufficiency [18] than any of the polymorphisms studied in isolation. While haplotypes represent genetically fixed combinations of polymorphisms within a single gene, this new concept is looking at functionally related polymorphisms (FRPs) [19]. However, the validity of this concept has not been tested against phenotypes other than those directly related to renal function. Against this background, we have explored whether the FRP concept indeed provides stronger/more consistent phenotype associations when applied to a different phenotype, i.e. HT. To increase the robustness of our approach, the high blood pressure phenotype was characterized concomitantly by the presence of HT or by systolic, diastolic or mean arterial pressure.

## Methods

### Study population

Our analysis is based on data obtained from the SUNSET study (Surinamese in The Netherlands: Study on Ethnicity and Health). In 1975, almost half the population of the former Dutch colony Surinam migrated to the Netherlands. Approximately 80% of these immigrants are black ("Creoles", a mix of African, European and other ethnic groups) or South Asian ("Hindustanis", originally from the Indian sub-continent). SUNSET is based on a random sample of 2975 individuals, aged 35-60 years, drawn from the Amsterdam population register, as previously described [20]. For further detailed description of the study population, sampling procedure and determination of ethnicity see also [21-23]. Following approval of the study protocol by the Medical Ethical Committee of the Academic Medical Center of the University of Amsterdam, all participants gave informed written consent. Subjects, who could not unequivocally be assigned to one of the three ethnic groups, who did not undergo a blood pressure measurement or who could not be genotyped were excluded from the present study, leaving a total of 1379 persons for our present analysis although not all variables could be documented for each of these subjects [23].

### Measurements

Blood pressure was measured in the morning on the right arm in a seated position with a validated, automated, oscillometric, digital device (OMRON M-4, Omron Healthcare, Kyoto, Japan) by trained staff using appropriate cuff sizes. Two readings were taken after the subject had emptied the bladder and had been seated for at least 5 min; the mean of the two readings was used in the analyses [21]. In line with our previous genotype/phenotype association studies [23], we operationally defined the high blood pressure phenotype as either the binary/categorical variable HT or the linear variables systolic blood pressure (SBP), diastolic blood pressure (DBP) and mean arterial pressure (MAP). HT was operationally defined as having a measured SBP >140 mmHg and/or a DBP >90 mmHg and/or taking antihypertensive drugs. All other subjects were assumed to have normal blood pressure.

### Genotyping

DNA was isolated from whole blood using an Autopure LS (Gentra Systems, Minneapolis, MN, USA). The AGT M235T (rs699), ACE I/D (rs4340) and AGTR1 A1166C (rs5186) polymorphisms were genotyped as previously described [24].

### Data analysis

Allele frequency was calculated in Haploview version 3.11 [25]. FRP analysis was performed with PHASE version 2.1 analogous to the analysis of haplotypes [26,27], which generates two FRPs per person. For FRP analysis we used double and triple combinations of the RAAS components.

Data on continuous variables are shown as means ± SD or as parameter estimates with 95% confidence intervals. Categorical variables such as the prevalence of alleles and FRPs as well as the prevalence of HT are reported as proportions (%). Differences in prevalence of HT between groups defined by genotype were tested using Fisher's exact test (Table 1). Multiple linear regression analysis, adjusted for gender, ethnicity, age, body-mass-index (BMI) and the presence of concomitant antihypertensive medication was used to study the association between SBP, DBP and MAP on the one and single polymorphisms or FRPs on the other hand (Table 2). Differences between groups are given as mean parameter estimate with 95% confidence intervals. For the AGT and ACE genes, the M and I alleles, respectively, were defined as the reference group as they should if anything associate with normotension because of their association with lower expression [6-8]. Due to limited functional information there is no clear biological way to define which of the AGTR1 gene alleles should be associated with normotension and hence be seen as the reference allele; as our initial analysis pointed to a lower incidence of HT with the C than with the A allele, we have operationally defined the C allele carriers as the reference group for our analysis. All statistical tests were two-tailed and p-values < 0.05 were considered statistically significant. All statistical analyses were done using SPSS 16.0 for Windows (SPSS Inc., Chicago, IL, USA) or Prism version 4.0 (GraphPad, San Diego, CA, USA). Post-hoc power calculations were performed using the G*Power3 program http://www.psycho.uni-duesseldorf.de/abteilungen/aap/gpower3/.

**Table 1:** Prevalence of alleles and FRPs.

Genotype	Number of alleles/FRPs	% prevalence of allele/FRP	Number of subjects with allele/FRP and HT	% prevalence of HT in group with allele/FRP
**AGT**				

M	954	36.5	287	30.1

T	1662	63.5	561	33.9 (0.0512)

**ACE**				

D	1352	51.3	451	33.5

I	1284	48.7	411	32.1 (0.4630)

AGTR1				

C	402	15.4	109	27.1

A	2208	84.6	739	33.6 (0.0126)

**AGT/ACE combination**				

M/I	385	12.9	107	30.1

T/D	764	27.7	270	35.3 (0.0560)

**AGT/AGTR1 combination**				

M/C	294	10.7	83	28.2

T/A	1590	57.7	548	34.5 (0.0372)

**ACE/AGTR1 combination**				

I/C	298	10.8	79	26.5

D/A	1253	45.4	420	33.5 (0.0227)

**AGT/ACE/AGTR1 combination**				

M/I/C	203	7.4	56	27.6

T/D/A	803	29.1	279	34.7 (0.0555)

Data are absolute numbers of alleles (2 alleles per person and 2 FRPs per person) and their relative prevalence in %. P-values from Fisher's exact test vs. the reference genotype (M, I and C alleles, respectively) for the % prevalence of HT are given in parentheses. Note that two alleles/FRPs were identified per subject.

**Table 2:** Difference in systolic blood pressure (SBP), diastolic blood pressure (DBP) and mean arterial pressure (MAP) associated with genotype.

	SBP	DBP	MAP
	**AGT polymorphism**		

MM	Reference group		

MT	-0.0 (-4.2; 4.2)	-0.6 (-3.1; 1.9)	-0.4 (-3.3; 2.5)

TT	2.4 (-1.9; 6.7)	0.8 (-1.8; 3.4)	1.3 (-1.7; 4.3)

	**ACE polymorphism**		

II	Reference group		

ID	-0.9 (-3.8; 1.8)	0.6 (-1.1; 2.2)	0.1 (-1.9; 1.9)

DD	0.2 (-3.1; 3.5)	0.5 (-1.5; 2.4)	0.4 (-1.9; 2.7)

	AGTR1**polymorphism**		

CC	Reference group		

CA	5.7 (-3.9; 15.4)	2.4 (-3.4; 8.1)	3.5 (-3.2; 10.2)

AA	8.2 (-1.3; 17.7)	4.2 (-1.5; 9.8)	5.5 (-1.1; 12.1)

	**Combined AGT/ACE polymorphism**		

M/I	Reference group		

T/D	0.1 (-3.1; 3.4)	0.6 (-1.3; 2.5)	0.4 (-1.8; 2.7)

	**Combined AGT/**AGTR1**polymorphism**		

M/C	Reference group		

T/A	2.3 (-0.1; 4.6)	1.3 (-0.1; 2.8)	1.6 (-0.0; 3.3)

	**Combined ACE/**AGTR1**polymorphism**		

I/C	Reference group		

D/A	2.1 (-0.1; 4.3)	1.4 (0.1; 2.7)	1.6 (0.1; 3.2)

	**Combined AGT/ACE/**AGTR1**polymorphism**		

M/I/C	Reference group		

T/D/A	2.7 (-0.1; 5.4)	1.7 (0.1; 3.3)	2.0 (0.1; 3.9)

Data are shown as differences relative to the respective reference group expressed in mm Hg (95% confidence intervals) adjusted for age, gender, body mass index, ethnicity and concomitant antihypertensive medication. Note that confidence intervals excluding 1 are statistically equivalent to a p < 0.05.

## Results

### Basic and genetic characteristics of the study population

The mean age of the study population (42.1% Blacks, 36.3% Whites, and 21.5% South Asians) was 45.4 ± 6.7 years with 41.6% being male. The BMI was 27.3 ± 5.2 kg/m^2^. Mean SBP, DBP and MAP were 126.3 ± 19.7 mmHg, 82.0 ± 11.6 mmHg and 96.8 ± 13.8 mmHg, respectively, with 15.6% of all subjects reporting use of antihypertensive medication. According to our categorical definition 451 subjects (32.7%) were hypertensive. The genotype prevalences are shown in Table 1. AGT T and AGTR1 A represented the major alleles, whereas ACE D and I were similarly prevalent. ACE I/D and AGTR1 A1166C polymorphisms were in Hardy-Weinberg equilibrium, whereas the AGT M235T SNP was not.

### Genotype association with HT

While the prevalence of HT did not differ significantly between carriers of the AGT or ACE alleles, a significantly higher HT prevalence was found in A allele carriers of the AGTR1 (33.6% vs. 27.1%; Table 1). Accordingly, FRPs containing the A allele of the AGTR1 also were associated with a higher prevalence of HT, whereas the FRP composed of alleles from AGT and ACE was not significantly associated with HT (Table 1). Post-hoc calculations showed that the power to detect a group difference in the prevalence of HT based on genotype was >0.6 for the AGT or AGTR1 polymorphisms in isolation and in combination. Given that the prevalence of HT was similar with both ACE alleles, the power for detecting a difference between them was low (<0.2). Accordingly, the triple combination had a power of 0.482 to detect a group difference.

### Genotype association with blood pressure

Linear regression models using gender, ethnicity, age, BMI and concomitant antihypertensive medication as covariates did not detect statistically significant or clinically relevant associations of AGT or ACE alleles with SBP, DBP or MAP (Table 2). Consequently, the FRP combining AGT and ACE alleles was also not associated with blood pressure differences. While the A allele of the AGTR1 was consistently associated with a numerically higher SBP, DBP and MAP and even showed an apparent gene dose-response, these differences did not reach statistical significance for any of the three blood pressure measurements (Table 2). FRPs including the A allele of the AGTR1 were also associated with higher SBP, DBP and MAP; while these associations reached statistical significance in several cases, they exhibited smaller effect sizes than the A allele in isolation (Table 2).

## Discussion

As AGT, ACE and AGTR1 act in concert, it has been proposed that combinations of polymorphisms in the corresponding three genes may exhibit stronger/more consistent associations with phenotypes related to renal function than those in each of the three genes studied in isolation [17,18]. The present analysis was designed to test this concept for a different phenotype, i.e. HT. However, our data do not support the idea that the FRP concept can be extended to the high blood pressure phenotype.

### Critique of methods

Our analysis originates from a population-based sample rather than a case-control design. This implies that only a fraction of all participants were hypertensive and that a fraction of the HT subjects were currently receiving antihypertensive treatment. A detailed description of this population has been presented previously [20-23].

While the PHASE program should also be suitable for FRP analysis on mathematical grounds, it should be pointed out that it was neither designed nor validated for such analysis.

The detection of genotype-phenotype associations may be complicated by various factors including ethnicity but despite a large number of previous studies there is no consistent evidence for ethnicity-dependent associations of RAAS component polymorphisms with HT [5,11]. As some ethnic groups were too small to allow analysis of multiple loci, we have used the entire database but included ethnicity as a co-explanatory variable to be on the safe side. Ethnic differences in HT prevalence are unlikely to have contributed to our analysis as this was the target phenotype. Nevertheless, we cannot exclude that a genotype may only manifest phenotypically in the context of a specific ethnicity, and it also has to be considered that polymorphisms may display an ethnicity-dependent prevalence [28,29].

To increase the robustness of our analysis [23,30], we have concomitantly looked at two definitions of high blood pressure phenotype, i.e. the presence of HT (categorical variable) and measured blood pressure (continuous variable). For the latter, SBP, DBP and MAP were used as target variables in order to further increase the robustness of the analysis. Our use of the categorical variable HT is in line with most previous studies but this is less straightforward than it may appear because factors such as age or obesity may confound the qualitative diagnosis of HT. Moreover, categorical variables tend to yield less statistical power than continuous variables. Therefore, we have concomitantly done all analyses based upon the linear variables of SBP, DBP and MAP. Possible confounding factors such as age, gender, ethnicity, BMI and concomitant antihypertensive medication were accounted for by using them as covariates in multiple regression models. Among these potential confounders, concomitant antihypertensive medications is most difficult to handle in a population-based settings as standardized pre-treatment blood pressure measurements typically are not available. Restriction of the analysis to control and non-treated hypertensive subjects was not done for two reasons. Firstly, this would introduce bias into the analysis which negates the advantages of a population-based sample. Secondly, as about half of our hypertensive subjects were on treatment (not atypical for a population-based sample), their elimination from the analysis would yield a too small group size for a meaningful FRP analysis. Our previous work has shown that the inclusion of concomitant medication as a covariate probably is, at least in this setting/situation, the most robust way of handling this confounder [23].

### Single gene polymorphisms

In our data set the M235T SNP in the AGT gene or the ID polymorphism in the ACE gene did not significantly associate with the blood pressure/hypertension phenotype. This is in line with the overall picture emerging from many previous studies looking at these polymorphisms [5] or the genome-wide studies [12-15]. While the AGT polymorphism in our data set did not exhibit Hardy-Weinberg equilibrium, this does not affect the overall picture that neither of these two polymorphisms in isolation has a major role for blood pressure despite their proven effect on the function of the RAAS. On the other hand, the A allele of the A1166C SNP within the AGTR1 gene was associated with a higher blood pressure in our analysis. While a large number of studies on this polymorphism did not reveal a major association with blood pressure, if anything the C allele of this SNP had been associated with higher blood pressure/hypertension [5,11]. Given the overall lack of association, the occurrence of a study with the A allele showing an association actually was not unexpected if indeed this locus has no major impact on the blood pressure/hypertension phenotype as suggested by the genome-wide studies [12-15].

### Single gene polymorphism vs. FRP associations with high blood pressure phenotypes

The main question of our analysis was whether the use of FRPs allows detection of stronger and/or more consistent detection of genotype/phenotype associations. With regard to the categorical variable HT, FRPs were only associated if they included the A allele of the AGTR1 gene. In these cases the prevalence of HT was rather similar in FRPs containing the A allele as compared to A allele carriers studied irrespective of their genotype for the two other genes. A more complex situation was found for the continuous phenotypic variables SBP, DBP and MAP. For these three variables the use of FRPs yielded statistical significance of genotype/phenotype associations in some cases where that was not found for the A allele studied in isolation. On the other hand, apparent effect sizes of the A allele were smaller when viewed in the context of FRPs than when looking at the A allele in isolation. Reaching statistical significance despite smaller group and smaller effect sizes indicates that the use of FRPs has yielded more consistent subpopulations with smaller intra-group variance.

## Conclusions

At least for the high blood pressure phenotype, our data do not support the previously proposed concept that FRPs yield stronger genotype/phenotype associations than individual polymorphisms of components of the RAAS. However, they may yield more homogenous patient groups, a possibility to be confirmed in future studies in independent populations. FRPs containing not only genes within the RAAS but also those of effectors of the RAAS, e.g. those involved in sodium and water handling, may also be worthwhile exploring. However, a more in-depth analysis of haplotypes within single genes and or the search for rare mutants with severe consequences may be a more promising approach.

## Abbreviations

ACE: angiotensin-converting enzyme; AGT: angiotensinogen; AGTR1: angiotensin II type 1 receptor; DBP: diastolic blood pressure; FRP: functionally related polymorphism; HT: hypertension; I/D: insertion/deletion; MAP: mean arterial pressure; RAAS: renin-angiotensin-aldosterone system; SBP: systolic blood pressure; SNP: single nucleotide polymorphism

## Competing interests

The authors declare that they have no competing interests.

## Authors' contributions

IGMvV and RPK were involved in the design of the SUNSET study. GM was involved in sample collection. INH, RPK and MCM were involved in the design of this analysis. All authors contributed to writing the manuscript, which has primarily been drafted by INH and MCM.

## Pre-publication history

The pre-publication history for this paper can be accessed here:

http://www.biomedcentral.com/1471-2261/10/23/prepub
